# Supplementary material for: Using mathematical modelling to investigate the adaptive divergence of whitefish in Fennoscandia
Source: Sci Rep. 2020 Apr 30;10:7394. doi: 10.1038/s41598-020-63684-3 (PMC7193591; doi:10.1038/s41598-020-63684-3)
Supplement: Supplementary file 1 — Supplementary information - appendix. [file 41598_2020_63684_MOESM1_ESM.pdf]

Supplementary information for: Using mathematical modelling to investigate the adaptive divergence of whitefish in Fennoscandia.

Monday 23rd March, 2020

Xavier Thibert-Plante[1], Kim Præbel[2], Kjartan Østbye[3,4], Kimmo K. Kahilainen[5], Per-Arne Amundsen[6], and Sergey Gavrilets[7]

[1]Previous affiliations: Department of Ecology and Environmental Science, and IceLab, Umeå University, Umeå, Sweden.

[2]Norwegian College of Fishery Science, UiT The Arctic University of Norway, N-9037 Tromsö, Norway. Phone: (+ 47) 776 46107

[3]Inland Norway University of Applied Sciences, Department of Forestry and Wildlife Management, Campus Evenstad, Anne Evenstadsvei 80, NO-2480 Koppang, Norway.

[4]Centre for Ecological and Evolutionary Synthesis (CEES), Department of Biosciences, University of Oslo, PO Box 1066 Blindern, NO-0316 Oslo, Norway.

[5]Lammi Biological Station, University of Helsinki, Pääjärventie 320, FI-16900 Lammi, Finland

[6]Department of Arctic and Marine Biology, Faculty of Biosciences, Fisheries and Economics, UiT The Arctic University of Norway, N-9037 Tromsö, Norway.

[7]Department of Ecology and Evolutionary Biology, Department of Mathematics, National Institute for Mathematical and Biological Synthesis (NIMBioS), Center for the Dynamics of Social Complexity (DySoC), University of Tennessee, Knoxville, TN 37996 USA.

Appendices

Demographic equilibrium

To estimate the carrying capacity parameters $K_{s,j}$ we proceeded in several steps. First, we estimated the birth rate for whitefish of different size using data in [43,44]. Second, using personal observations on the proportions of mature fish in each niche for trimorphic lakes we came up with realistic values of their equilibrium densities (N) at 1000, 6000 and 150 individuals, for littoral, pelagic and profundal morphs, respectively. These values serve as default values in our simulations.

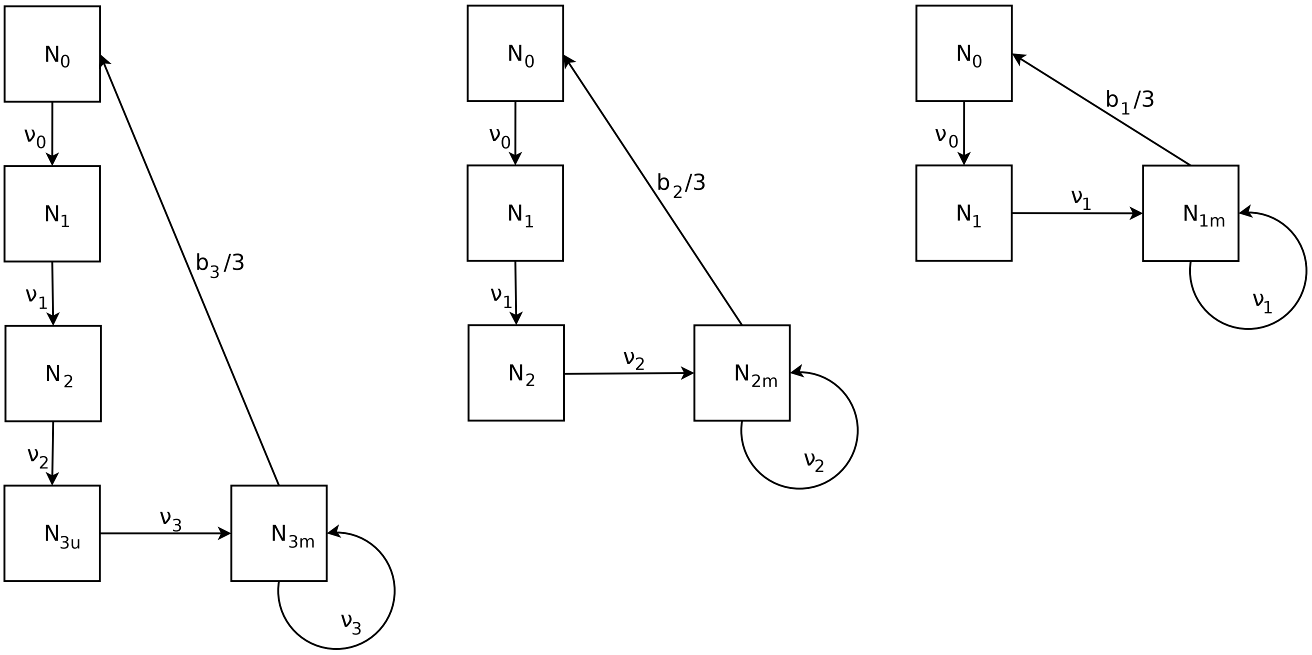

Figure S1: Demographic analysis of each population. N_s, ν_s and b_s are the population density, survival rate, and fertility at size s, respectively. Subscripts u and m specify immature and mature individuals.

Then, using the Leslie matrices approach (see Figure S1) and assuming perfect adaptation and no predation, we find that the equilibrium population densities N_s of fish of different size in

different niches must satisfied the following equalities:

$$\begin{array}{lll} \text{Littoral} & \text{Profundal} & \text{Pelagic} \\ N_0 = \frac{N_{3m} b_3}{3} & N_0 = \frac{N_{2m} b_2}{3} & N_0 = \frac{N_{1m} b_1}{3} \\ N_1 = N_0 v_0 & N_1 = N_0 v_0 & N_{1u} = N_0 v_0 \\ N_2 = N_1 v_1 & N_{2u} = N_1 v_1 & N_{1m} = v_1 (N_{1u} + N_{1m}) \\ N_{3u} = N_2 v_2 & N_{2m} = v_2 (N_{2u} + N_{2m}) & \\ N_{3m} = v_3 (N_{3u} + N_{3m}) & & \end{array} \tag{S1}$$

where b_s is the average number of offspring per female of size s, and subscripts u and m denote immature and mature individuals, respectively. Note that the division by three in all equations for N_0 above is due to the 2:1 male:female sex ratio in whitefish (Author personal observations).

Littoral morph. Exact data needed to estimate all parameters of our model are difficult to come up with. As a result in some cases we have to take an educated guess. Using our unpublished data (Author unpublished), we know that one of the $2,048$ sampled littoral fish survived up to 26 years, thus living 23 years at stage three. This gives the yearly survival rate of $v_3 \approx 0.71$. We then can find a relation between the mature and immature population sizes at stage three. With $N_3 = 1000$, we have $N_{3m} = 709$ and $N_{3u} = 291$. From Eq. 2 in the main text, we calculate $K_{s,j}$:

$$K_{s,j} = \frac{N_{s,j} v_{s,j}(x)}{\omega_{s,j}(x)(1 - v_{s,j}(x))} \tag{S2}$$

Assuming adapted individuals ($\omega_{s,j}(x) = 1$), we can now calculate the carrying capacity parameter $K_3 = \frac{v_3 N_3}{1 - v_3} = 2448$. Assuming that $b_3 = 64$, from Eq. S1 we get the number of stage zero individuals in the littoral environment $N_0 = 15125$.

We now have to set the other three survival rate v_0, v_1 and v_2. We know their product because it is equal to the ratio of stage 3 and 0 individuals: $v_0 v_1 v_2 = \frac{N_{3u}}{N_0} = 0.019$. One possible simple combination of surviving rates that appears to be realistic is $(v_0, v_1, v_2) = (0.2, 0.25, 0.4)$. With these values, we have equilibrium densities at $(N_0, N_1, N_2, N_{3u}, N_{3m}) = (15125, 3025, 756, 291, 709)$. Using equation S2, the carrying capacity parameters for sizes 0, 1 and 2 for the littoral morph become $K_0 = 3781, K_1 = 1008, K_2 = 504, K_3 = 2448$.

Stage	Littoral	Pelagic	Profundal
0 (2cm)	0.2/3781	0.338/2817	0.1/73
1 (15cm)	0.25/1008	0.69/13355	0.4/44
2 (20cm)	0.4/504	0/0	0.82/683
3 (30cm)	0.71/2448	0/0	0/0

Table S1: Survival $v_{s,j}$/carrying capacity $K_{s,j}$ at equilibrium for the different stage in different habitats.

Profundal morph. In order to find parameters for the carrying capacity of the produndal population, we need to make further assumptions: First, we set its fertility at $b_2 = 16$ which fits the known relationship between fish size and the number of eggs produced [43,44]. In our unpublished data, of the 243 profundal whitefish sampled, the oldest one was 30 years old. Using the same logic as above, we find its survival rate $v_2 = 0.82$ at stage two. We set the equilibrium density of the largest profundal fish to $N_2 = 150$. Using those assumptions we get a profundal niche with the following equilibrium densities $(N_0, N_1, N_{2u}, N_{2m}) = (656, 66, 123, 27)$ and survival $(v_0, v_1, v_2) = (0.10, 0.40, 0.82)$. Using equation S2, the carrying capacity parameters for sizes 0, 1, and 2 for the profundal morph become $K_0 = 73, K_1 = 44, K_3 = 683$.

Pelagic morph. To extract the survival and carrying capacities of the pelagic population, we again use the relationship between fish fertility and size [43,44] and get $b_1 = 4$. Of the 913 profundal whitefish sampled, five reached the age of 16 years old. Using the same logic as above, we have a survival rate of $v_1 = 0.69$ at stage one. In this case, we use $N_1 = 6000$, and $v_1 = 0.69$ and extract the other parameters using the Leslie equations above. We now have the equilibrium densities $(N_0, N_{1u}, N_{1m}) = (5517, 1862, 4138)$ and survival $(v_0, v_1) = (0.338, 0.69)$ for the pelagic niche. Using equation S2 results in carrying capacity parameters $K_0 = 2817, K_1 = 13355$ for the pelagic environment.

Table S1 summarizes the estimates of equilibrium survival rates v and K parameters.
